# Trajectory of test components of a comprehensive geriatric assessment in orthogeriatric patients following total hip and knee arthroplasty

**DOI:** 10.1186/s12877-026-07307-y

**Published:** 2026-03-11

**Authors:** Tobias Kappenschneider, Philip Bammert, Dominik Emanuel Holzapfel, Julia Sabrina Schiegl, Stefano Pagano, Loreto C Pulido, Joachim Grifka, Katrin Michalk, Jan Reinhard

**Affiliations:** 1 Department of Orthopaedics, Regensburg University, Kaiser-Karl V. Allee 3, Bad Abbach, 93077 Germany; 2 Department of Health Economics, Technical University of Munich, Munich, Germany; 3 Department of Orthopaedics and Trauma Surgery, Fichtelgebirge Medical Center, Marktredwitz, Germany; 4 Research Centre for Orthopaedics and Ergonomics, East Bavarian Technical University of Applied Sciences Regensburg, Regensburg, Germany

**Keywords:** Comprehensive Geriatric Assessment (CGA), Total hip arthroplasty (THA), Total knee arthroplasty (TKA), Orthogeriatric, Rehabilitation, Frailty

## Abstract

**Background:**

A comprehensive geriatric assessment (CGA) is considered the gold standard for improving numerous relevant endpoints for older people in hospitals. The aim of the study was to investigate what happens across time to individual test components of a CGA after completion of inpatient rehabilitation and 3 months after surgery compared to the start of rehabilitation in orthogeriatric patients who underwent elective total hip or knee arthroplasty (THA or TKA).

**Methods:**

In this prospective study, we used data from 186 participants (THA *n* = 110, TKA *n* = 76) of the Special Orthopaedic Geriatrics (SOG) trial, funded by the German Federal Joint Committee (GBA). Study data were collected preoperatively (basic information), on admission to inpatient rehabilitation (7th postoperative day), on discharge (4 weeks postoperatively) and 3 months after THA or TKA. Non-parametric Wilcoxon signed rank tests were used for the main statistical analyses, and Mann-Whitney U tests were employed for secondary analysis stratified by age, frailty, and mobility.

**Results:**

Compared to admission, both surgical groups showed significant improvements in Barthel Index, Nutritional Risk Screening (NRS), frailty, Short Physical Performance Battery (SPPB), gait speed and reduction in polypharmacy on discharge from inpatient rehabilitation (effect size, 0.18–0.79). 3 months postoperatively, there were further significant improvements in Barthel Index, SPPB, gait speed and reduction in polypharmacy in both groups compared to discharge from rehabilitation (effect size, 0,38 − 0,75), while NRS and frailty only improved in the THA group. Differentiated according to the presence of frailty (Fried’s frailty criteria ≥ 3), both groups demonstrated significantly better improvements in frail participants than in robust or pre-frail patients.

**Conclusion:**

The results of this study reveal that key components of CGA significantly improve in orthogeriatric patients after THA and TKA three weeks after inpatient rehabilitation and continue to improve up to three months after surgery. There is evidence that obviously frail patients benefit particularly well from rehabilitation programmes.

**Trial registration:**

This study is part of the Special Orthopaedic Geriatrics (SOG) trial, German Clinical Trials Register DRKS00024102. Registered on 19 January 2021.

## Background

Osteoarthritis (OA) affects more than half of the global population aged 65 and older [1, 2]. The prevalence of OA increases inexorably with age, as the disease is irreversible. Hip and knee OA are major causes of immobility, especially in older people. Sarcopenia, frailty, morbidity, loss of activities of daily living (ADLs) and reduced quality of life may also be associated with OA [1, 3]. A primary, elective total hip arthroplasty (THA) or knee arthroplasty (TKA) is often the only way to regain mobility and improve quality of life after all conservative therapies have been exhausted. The number of primary hip and knee arthroplasty surgeries worldwide has increased by 30% and 40% respectively over the last two decades due to demographic trends [4]. In the US, THA is expected to increase dramatically by 176% by 2040 and 659% by 2060, and TKA by 139% by 2040 and 469% by 2060 [5]. Older patients are known to have an increased risk of postoperative complications and mortality in hip and knee arthroplasty surgeries [6, 7]. However, recent studies show that they significantly benefit from total joint arthroplasty (TJA) in terms of functional outcomes [8, 9]. In Germany, a THA or TKA and a stay in an orthopaedic hospital are usually followed by three weeks of inpatient rehabilitation for orthogeriatric patients. The aim of inpatient rehabilitation is to improve physical activity, ADLs, social participation, quality of life and to prevent the need for long-term care [10–12]. The World Health Organisation (WHO) defines the maximisation of function and the minimisation of activity and participation restrictions caused by an impairment or disease as the primary goals of rehabilitation [13].

In orthogeriatrics, the focus of scientific research to date has been primarily on patients with hip fractures. And even here, there are only a few studies in the literature that analyse the course of rehabilitation with the inclusion of a comprehensive geriatric assessment (CGA) [14]. Less is known about the course of CGA in geriatric and multimorbid patients after total hip and knee arthroplasty and subsequent inpatient rehabilitation. The CGA is considered the gold standard for improving numerous relevant endpoints for older people in hospitals. It is a multi-dimensional, multi-disciplinary diagnostic and therapeutic process conducted to determine the medical, mental, and functional problems of older people with frailty so that a co-ordinated and integrated plan for treatment and follow-up can be developed [15]. CGA has the potential to improve health-related outcomes while reducing health and social care costs [16]. It is also well suited for evidence-based monitoring of the clinical progress of a treatment [15].

The aim of the study was to evaluate what happens across time to individual test components of a CGA after completion of inpatient rehabilitation and 3 months after surgery compared to the start of rehabilitation in orthogeriatric patients undergoing elective THA and TKA. In terms of the main analyses, we hypothesised that the individual test components of the CGA would improve after completion of rehabilitation and that these positive effects would be maintained or even increase over time.

## Methods

### Study design

This study is part of the Special Orthopaedic Geriatrics (SOG) trial (German Clinical Trials Register, 19/01/2021, DRKS00024102). The SOG study is a monocentric, prospective, randomised controlled trial funded by the German Federal Joint Committee (GBA). The original study aimed to evaluate a specially developed multimodal care model (SOG care model) for orthogeriatric hip and knee replacement patients compared with standard orthopaedic care without orthogeriatric co-management during inpatient orthopaedic hospitalisation. A comprehensive geriatric assessment was performed prospectively at multiple time points. A detailed description of the study can be found elsewhere [17]. Between 1 April 2021 and 31 January 2024, a total of 203 patients from the SOG trial were enrolled in the current study. This additional analysis was not planned when the original study was designed.

### Data collection

In the Orthopaedics Department of the Regensburg University Medical Centre, about 18,000 patients are treated annually in the university outpatient clinic and > 1,500 endoprosthetic procedures on hip and knee joints are performed each year. Participants were recruited at the university outpatient clinic if they were diagnosed with primary hip or knee osteoarthritis and had an indication for THA or TKA. Postoperatively, the patients remained in hospital for 7 days, unless complications occurred. All patients were mobilised under full weight bearing immediately after surgery. They received daily physiotherapy and were then discharged to inpatient rehabilitation. Study data were collected preoperatively (basic information), on admission to inpatient rehabilitation (7th postoperative day), on discharge (4 weeks postoperatively) and 3 months after TJA. Data collection was carried out for all patients and at all observation points of the study by a geriatric-trained study team in the university outpatient clinic. Patients were given additional appointments for this and were summoned to the university hospital. The medications were determined by the geriatrician based on patient reports or the federal standard medication plan. A research assistant, physiotherapist, occupational therapist and geriatrician carried out the test procedures. Data collection and CGA were conducted under the strict supervision of a geriatric investigator. The study team was trained in working with geriatric patients and in performing CGA, and routinely conducts geriatric testing procedures in everyday clinical practice. All geriatric testings of the CGA were implemented in accordance with their specifications and licensing. The CGA was performed digitally using the ORBIS AddOn GERD from Dedalus Health Care. All necessary licences were supplied by the medical software provider [17].

### Study population

The prevailing analysis was performed on a subsample of the total SOG study population, for which an a priori sample size calculation was performed using G*Power (Version 3.1.9.7). The calculation was based on a two-sided significance level of α = 0.05 and statistical power of 0.80. An effect size of 0.5 was conservatively assumed based on the existing literature on the topic and pilot date. Additionally, a drop-out rate of 20% was assumed [17]. Eligibility criteria included: primary hip or knee osteoarthritis, age ≥ 70 years and multimorbidity or age ≥ 80 years and indication for elective unilateral hip or knee replacement. Exclusion criteria were age < 70 years, previous bony surgery or tumour in the area of the joint to be treated, acute infection and increased need for care (care level ≥ 4; severe impairment of independence, need for help with basic care 24 h a day) [17].

### Surgical techniques and implants

All operations were performed in a single Department of Orthopaedic Surgery of a University Medical Centre. The lateral decubitus position was used for the THA. A minimally invasive anterolateral approach was chosen [18]. Press-fit acetabular components and stems from a single manufacturer (Pinnacle cup, Corail stem; DePuy, Warsaw, IN, U.S.) were used in all THAs. Cementless stems were preferred. Knee arthroplasty was performed via a medial parapatellar approach. Cemented components from a single manufacturer (PFC Sigma; DePuy, Warsaw, IN, U.S.) were used in all TKAs. Patella resurfacing was not performed [17].

### Inpatient rehabilitation

Over 70% of patients were transferred to the rehabilitation facility affiliated with the hospital. The rehabilitants received individual therapy programmes and were treated by multidisciplinary teams (doctors, nurses, physiotherapists, masseurs, sports therapists, occupational therapists, psychologists, dieticians and team-integrated social workers). The duration of the inpatient rehabilitation programme was three weeks. The patient underwent either orthopaedic or geriatric rehabilitation.

### Comprehensive geriatric assessment (CGA)

The *Barthel Index* is an ordinal scale employed to measure performance in activities of daily living (ADL). A total of ten variables, which describe activities of daily living (ADL) and mobility, are scored. A higher number is indicative of a greater ability to function independently following hospital discharge [19]. The *Mini Mental State Examination (MMSE)* is a 30-point questionnaire extensively used in clinical and research settings to measure cognitive impairment. It examines functions such as registration (repeating named prompts), attention and calculation, recall, language ability, the ability to follow simple commands, and orientation. A score of 24 or more out of 30 indicates normal cognition. Scores below this can indicate severe (≤ 9 points), moderate (10–18 points) or mild (19–23 points) cognitive impairment [20]. The 15-item *Geriatric Depression Scale (GDS-15)* is a short version of the GDS. It is used to identify depression in older people. For each answer that indicates depression, the GDS gives you 1 point. A score of more than 5 out of 15 on the test may indicate depression [21]. *Nutritional Risk Screening (NRS)* is used to identify the presence of malnutrition and the risk of malnutrition in a hospital setting. It comprises four questions as a preliminary screening. If any of these questions are answered positively, a screening follows that includes substitute measurements of nutritional status using static and dynamic parameters as well as data on the severity of the disease (stress metabolism). A score of 0 to 3 can be assigned for each parameter. Being over 70 years of age is considered a basic risk factor, for which 1 point is automatically added. A total score of 3 or more points means that the patient is at risk of malnutrition or is already malnourished [22]. Frailty was assessed using the five criteria of the *Physical Frailty Phenotype* described by Fried [23, 24], which were adjusted as follows: shrinking (self-reported unintentional weight loss of more than 4,5 kg in the past year), exhaustion (self-reported using the CES-D depression scale), slowed gait speed (walking time of 5 m below an adjusted cut-off by gender and height), weakness [grip strength below an established cut-off based on gender and body mass index (BMI) measured on the dominant hand using a dynamometer (Jamar^®^ Hydraulic Hand Dynamometer; Performance Health, Wisconsin)] and low physical activity (kilocalories per week below an established gender-specific cut-off using self-reported frequency and duration of walking or cycling based on activity level according to the Swiss Health Observatory). Each criterion was scored as 0 or 1, depending on whether it was present or not. Robust patients were defined as having a score of 0, pre-frail patients as having a score of 1–2, and frail patients as having a score of 3 and higher. Physical performance was measured using the *Short Physical Performance Battery (SPPB)*. The SPPB test assesses balance, mobility, and muscle strength. It involves examining an ability to stand in different positions, the time taken to walk 4 m, and the time taken to rise from and sit on a chair five times. Scores for the tests range from 0 to 4, with a maximum total of 12 [25].

### Statistical analysis

Descriptive statistics including demographic and morbidity-related characteristics were calculated for TKA and THA patients. The distribution of all outcome measures was assessed graphically (histograms and Q–Q plots) and using the Shapiro–Wilk test. As the outcomes showed deviations from normality, nonparametric methods were applied; therefore, as a core statistical method, the non-parametric Wilcoxon signed rank test was utilised, which is prominently used for repeated measurements in a single sample. Inferential statistics were also calculated separately for TKA and THA patients. It was analysed whether there were significant differences between the start (t0) and discharge from rehabilitation (t1) as well as between discharge from rehabilitation (t1) and follow-up 3 months after surgery (t2). As outcomes, the measures described above were used. Means and standard deviations were reported for each time of measurement accompanied by the information whether there was a significant difference between two times of measurement and a corresponding effect size. Further procedures included subgroup analyses, in which the two samples (knee and hip patients) were further divided based on the patients’ age, frailty and physical performance measured by SPPB. It was then assessed whether the differences between t0 and t1 as well as t1 and t2 in the included outcome measures differed significantly between patients, that are either over or under a certain age-, frailty-, or SPPB-threshold. For this, the Mann-Whitney U test was employed. All analyses were conducted in R version 4.2.1. using the R-package “rstatix”. P-values of < 0.05 were considered statistically significant.

## Results

Out of a total of 203 patients in the SOG study, there were 17 drop-outs (7 patients with TKA and 10 patients with THA). The reasons were cancellation of the operation, non-admission to inpatient rehabilitation or refusal to participate in the study. As a result, the number of people included in the analysis was 186 (TKA *n* = 76, THA *n* = 110). In 5 participants with TKA and in 6 participants with THA, no CGA could be carried out on discharge from inpatient rehabilitation. The number of patients lost to follow-up after 3 months was 5 for the knee patients and 5 for the hip patients. The mean age of all patients was almost 80 years. Females were more frequently represented than males. The majority of participants in both the TKA and THA groups were pre-adipose, non-smokers and had an average of more than 7 comorbidities. Over 80% of all patients had hypertension. The most common other comorbidities included hyperlipidaemia, obesity, diabetes mellitus, chronic heart failure, cardiac arrhythmia and coronary heart disease as further cardiovascular risk factors. More than 63% of the TKA patients and over 55% of the THA patients suffered from polyarthrosis. The mean number of pre-existing medications was 9.8 in the TKA group and 10 in the THA group. Around 90% of the participants in the study underwent inpatient orthopaedic rehabilitation. Further details are presented in Table 1.

**Table 1:** Characteristics of patients who underwent total knee arthroplasty or total hip arthroplasty

	TKA patients (*n* = 76)	THA patients (n = 110)	
	mean (SD) or *n* (%)	mean (SD) or n (%)	
Gender, female	51 (67.1%)	69 (62.7%)	
Age, years	78.7 (4.9)	77.8 (4.8)	
Weight, kg	81.0 (15.5)	78.1 (15.2)	
BMI, kg/m²	29.5 (4.5)	28.5 (5.2)	
Care recipient	14.5%	13.6%	
Smoking	2.6%	5.5%	
CCI	5.2 (1.5)	5.3 (2.0)	
Comorbidities, n	7.6 (3.0)	7.3 (3.3)	
Hypertension	62 (81.6%)	91 (82.7%)	
Polyarthrosis	48 (63.2%)	61 (55.5%)	
Hyperlipidaemia	37 (48.7%)	49 (44.6%)	
Obesity	36 (47.37%)	41 (37.27%)	
Chronic renal insufficiency	25 (32.9%)	38 (34.6%)	
Diabetes mellitus	27 (35.5%)	30 (27.3%)	
Chronic heart failure	14 (18.4%)	20 (18.2%)	
Cardiac arrhythmia	20 (26.3%)	27 (24.6%)	
Hearing impairment	20 (26.3%)	22 (20.0%)	
Coronary heart disease	11 (14.5%)	22 (20.0%)	
Tumour disease	9 (11.8%)	18 (16.4%)	
Stroke	9 (11.8%)	10 (9.1%)	
Rheumatoid arthritis	5 (6.6%)	12 (10.9%)	
Medications, n	9.8 (3.9)	10.0 (4.1)	
In-house rehabilitation	57 (75.0%)	80 (72.7%)	
External rehabilitation	19 (25,0%)	30 (27,3%)	
Orthopaedic rehabilitation	68 (89,5%)	101 (91,8%)	
Geriatric rehabilitation	8 (10.5%)	9 (8.2%)	

*TKA* Total knee arthroplasty, *THA* Total hip arthroplasty, *SD* Standard deviation,* BMI* Body Mass Index, *CCI* Charlson Comorbidity Index

Table 2 compares the results of the CGA between the beginning and discharge from inpatient rehabilitation. Both the TKA and THA groups showed significant improvements in Barthel Index, NRS, frailty score, SPPB and gait speed after 3 weeks of rehabilitation. During this period, the number of postoperative medications was also significantly reduced from a mean of 9.8 (3.9) to 8.8 (3.4) for knee patients and from a mean of 10.0 (4.1) to 8.7 (4.0) for hip patients.

**Table 2:** Results of outcome measures at the beginning of rehabilitation and afterwards

	TKA patients (n = 76)			THA patients (n = 110)		
	mean (SD)		ES	mean (SD)		ES
	Admission	Discharge		Admission	Discharge	
Barthel Index	83.8 (10.8)	95 (10.2)*	0.79	84.7 (9.4)	92.7 (15.3)*	0.65
MMSE	27.6 (2.4)	27.7 (2.6)	0.06	27.8 (2.7)	27.5 (3.9)	0.02
GDS	2.3 (1.9)	2.1 (2.2)	0.21	2.5 (2.6)	2.5 (2.7)	0.04
NRS	1.4 (0.7)	1.1 (0.5)*	0.35	1.3 (0.6)	1.2 (0.6)*	0.18
Frailty	1.8 (1.4)	1 (1.3)*	0.55	2.1 (1.5)	1.4 (1.4)*	0.59
SPPB	6.3 (2.7)	7.5 (2.8)*	0.55	7.1 (2.8)	8.0 (3.0)*	0.40
Grip strength	27.7 (9.2)	28 (10.3)	< 0.01	27.4 (10.5)	27.3 (9.5)	0.05
Gait Speed	0.6 (0.2)	0.8 (0.3)*	0.74	0.6 (0.3)	0.8 (0.3)*	0.75
Medication, n	9.8 (3.9)	8.8 (3.4)*	0.52	10.0 (4.1)	8.7 (4.0)*	0.50

*TKA *Total knee arthroplasty, *THA* Total hip arthroplasty, *SD* Standard deviation, *ES* Effect size, *MMSE* Mini Mental Status Examination,* GDS* Geriatric Depression Scale (15-item version); *NRS* Nutritional Risk Screening,* Frailty* Fried’s frailty criteria, *SPPB* Short Physical Performance Battery, Grip strength in kg, 5-meter gait speed test in m/s

* = p < 0.05

Wilcoxon-signed-rank test

Table 3 describes the further course of the CGA between the end of rehabilitation and 3 months after TJA. Both groups showed significant improvements in Barthel Index, SPPB and gait speed. THA patients also had a further significant improvement in NRS and a further significant regression in frailty. Polypharmacy also continued to be significantly reduced in both groups.

**Table 3:** Results of outcome measures after rehabilitation and 3 months after surgery

	TKA patients (n = 71)			THA patients (n = 104)		
	mean (SD)		ES	mean (SD)		ES
	Discharge	3 mo. post-op		Discharge	3 mo. post-op	
Barthel Index	95 (10.2)	97.2 (8.2)*	0.38	92.7 (15.3)	98.0 (5.5)*	0.56
MMSE	27.7 (2.6)	27.8 (2.6)	0.08	27.5 (3.9)	28.0 (2.3)	0.03
GDS	2.1 (2.2)	2.3 (2.4)	0.12	2.5 (2.7)	2.4 (3.0)	0.12
NRS	1.1 (0.5)	1.2 (0.6)	0.07	1.2 (0.6)	1.0 (0.3)*	0.19
Frailty	1.0 (1.3)	0.8 (1.2)	0.14	1.4 (1.4)	0.8 (1.1)*	0.48
SPPB	7.5 (2.8)	8.6 (2.6)*	0.66	8.0 (3.0)	9.2 (2.3)*	0.52
Grip strength	28 (10.3)	28 (10.4)	0.11	27.3 (9.5)	27.4 (9.0)	0.04
Gait Speed	0.8 (0.3)	0.9 (0.3)*	0.65	0.8 (0.3)	1.0 (0.3)*	0.62
Medication, n	8.8 (3.4)	7.7 (3.4)*	0.75	8.7 (4.0)	8.1 (4.1)*	0.61

*TKA* Total knee arthroplasty,* THA* Total hip arthroplasty, *SD*  Standard deviation, *ES* Effect size, *mo.* month,* MMSE* Mini Mental Status Examination,* GDS* Geriatric Depression Scale,* NRS* Nutritional Risk Screening,* Frailty*  Fried’s frailty criteria,* SPPB*  Short Physical Performance Battery; Grip strength in kg, 5-meter gait speed test in m/s

* = p < 0.05

Wilcoxon-signed-rank test

**Fig. 1:**
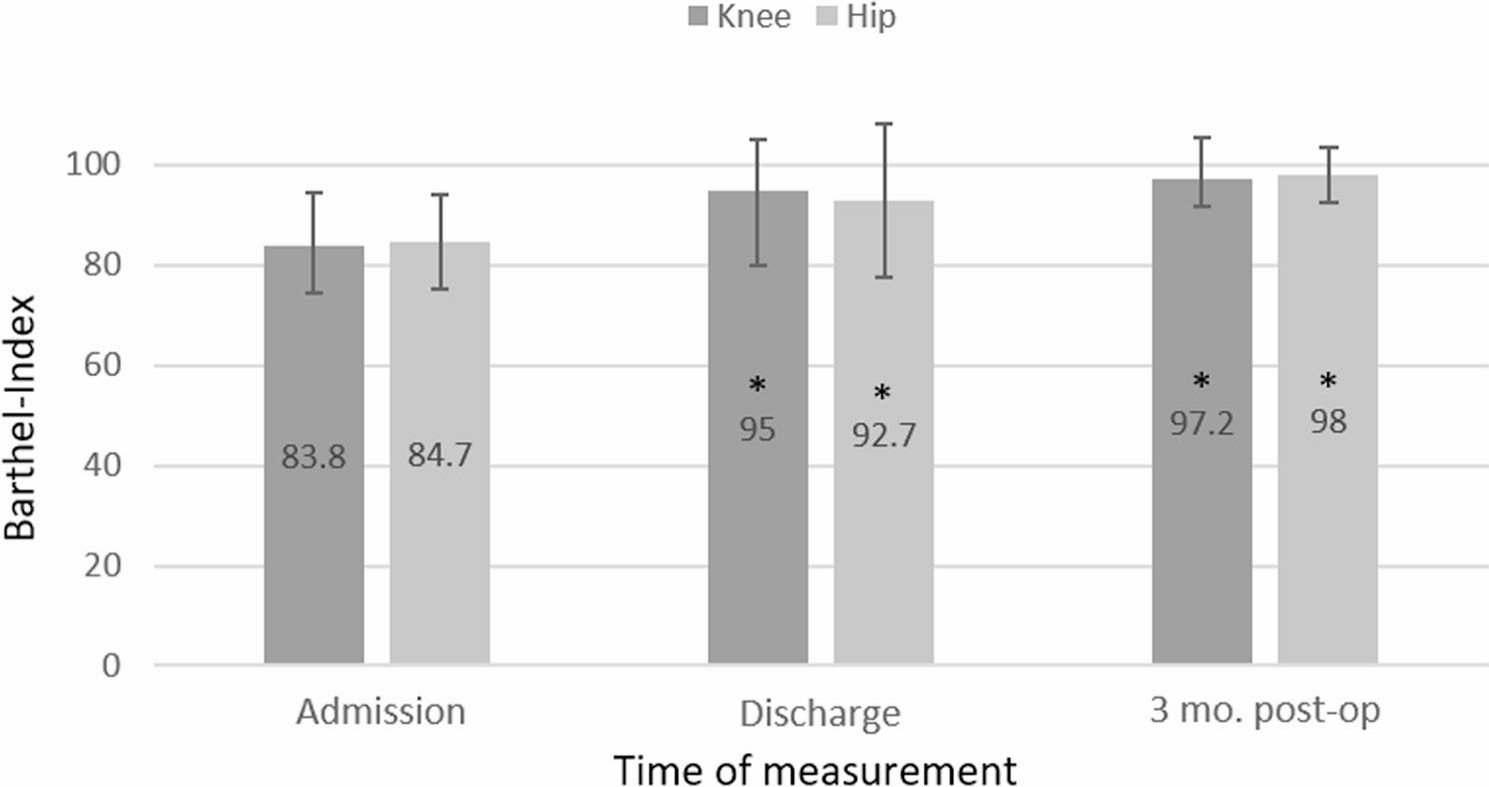
Barthel Indices (mean and SD) at the start and discharge of rehabilitation and 3 months after TJA

**Fig. 2:**
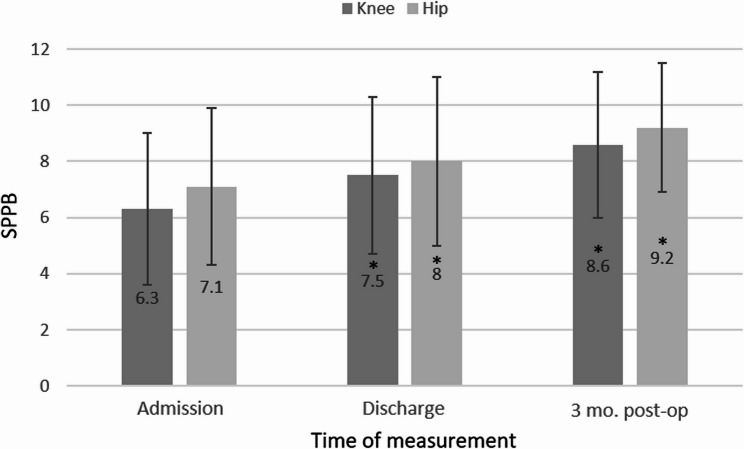
SPPB (mean and SD) at the start and discharge of rehabilitation and 3 months after TJA

The overall development of the Barthel Index and the SPPB is shown graphically in Figures 1 and 2.

Using the cut-off point of 80 years, the effects of rehabilitation were analysed with a delta score by subtracting the results at admission from those at discharge and the results at discharge from those 3 months after TJA. Patients in the TKA group who were 80 years of age or older had poorer improvements in gait speed between discharge and admission compared to patients under 80 years of age. Apart from this, there were no other significant differences based on age (Table 4).

**Table 4:** Differences between start and end of rehabilitation as well as end of rehabilitation and 3 months after surgery based on age

Differences between Admission and Discharge (Δ = Discharge - Admission)				
	TKA patients (n = 71), mean (SD)		THA patients (n = 104), mean (SD)	
Age, years	< 80	≥ 80	< 80	≥ 80
	*n* = 38	*n* = 38	*n* = 66	*n* = 44
Barthel Index	12.2 (7.2)	10.0 (12.0)	6.9 (14.4)	9.2 (11.7)
MMSE	-0.2 (1.6)	0.3 (2.2)	-0.3 (3.3)	0 (2.0)
GDS	-0.2 (1.4)	-0.2 (2.2)	0.1 (2.0)	-0.1 (1.5)
NRS	-0.2 (0.6)	-0.3 (0.9)	-0.1 (0.7)	-0.1 (0.5)
Frailty	-1.1 (1.2)	-0.6 (1.3)	-0.9 (1.2)	-0.6 (1.4)
SPPB	1.5 (1.6)	1.0 (2.4)	0.9 (2.9)	1.2 (2.1)
Grip strength	1.1 (4.6)	-0.4 (2.2)	-0.3 (3.5)	0.6 (3.2)
Gait Speed	0.3 (0.2)	0.2 (0.3)*	0.3 (0.2)	0.2 (0.2)
Medication, n	-1.3 (2.0)	-0.9 (2.0)	-1.1 (2.0)	-1.3 (2.7)

**Differences between Discharge and 3-mo. follow-up (Δ = 3 mo. post-OP - Discharge)**				
	TKA patients (n = 71), mean (SD)		THA patients (n = 105), mean (SD)	
Age, years	< 80	≥ 80	< 80	≥ 80
	*n* = 38	*n* = 38	*n* = 66	*n* = 44
Barthel Index	1.1 (4.6)	3.3 (10.3)	5.7 (16.7)	4.8 (7.3)
MMSE	0.1 (1.5)	0.2 (1.7)	0.3 (3.2)	0.1 (2.0)
GDS	0.1 (1.3)	0.2 (1.5)	-0.2 (2.0)	0.1 (1.5)
NRS	0.1 (0.8)	0 (0.8)	-0.2 (0.7)	0 (0.4)
Frailty	-0.1 (1.0)	-0.2 (0.9)	-0.6 (1.0)	-0.5 (1.0)
SPPB	1.0 (1.2)	1.5 (2.0)	1.3 (1.8)	1 (2.1)
Grip strength	-0.6 (3.6)	-0.2 (2.4)	0.7 (4.1)	-0.3 (3.4)
Gait Speed	0.1 (0.2)	0.2 (0.3)	0.2 (0.2)	0.1 (0.2)
Medication, n	-1.0 (0.9)	-1.1 (1.7)	-0.8 (1.6)	-0.7 (0.9)

*TKA *Total knee arthroplasty, *THA* Total hip arthroplasty, *SD* Standard deviation, *MMSE*  Mini Mental Status Examination, *GDS*  Geriatric Depression Scale, *NRS*  Nutritional Risk Screening, *Frailty*  Fried’s frailty criteria, *SPPB*  Short Physical Performance Battery, Grip strength in kg, 5-meter gait speed test in m/s

*=*p* < 0.05

Mann-Whitney-U test

Differentiated according to the presence of frailty (Fried’s frailty criteria ≥ 3), both TJA groups showed significantly better improvements in frail participants than in robust or pre-frail patients. This was the case for differences at the beginning and end of rehabilitation, as well as at discharge and 3 months after surgery (Table 5).

**Table 5:** Differences between start and end of rehabilitation as well as end of rehabilitation and 3 months after surgery based on frailty

Differences between Admission and Discharge (Δ = Discharge - Admission)				
	TKA patients (n = 71), mean (SD)		THA patients (n = 104), mean (SD)	
Frailty	< 3	≥ 3	< 3	≥ 3
	*n* = 49	*n* = 27	*n* = 61	*n* = 48
Barthel Index	9.3 (9.4)	14.6 (9.9)*	10.3 (7.2)	4.8 (17.8)
MMSE	0.1 (2.1)	0.1 (1.5)	-0.4 (1.6)	0.1 (3.8)*
GDS	-0.2 (2.1)	-0.2 (1.2)	0.1 (1.4)	0 (2.2)
NRS	-0.1 (0.4)	-0.6 (1.1)*	0 (0.6)	-0.2 (0.7)*
Frailty	-0.4 (0.9)	-1.6 (1.6)*	-0.3 (1.0)	-1.4 (1.2)*
SPPB	1.1 (1.9)	1.4 (2.3)	0.7 (2.2)	1.4 (3.0)*
Grip strength	0.3 (2.4)	0.6 (5.2)	-0.3 (2.9)	0.5 (3.9)
Gait Speed	0.2 (0.3)	0.3 (0.3)	0.2 (0.2)	0.3 (0.2)*
Medication, n	-0.8 (2.0)	-1.8 (1.8)	-1.3 (1.6)	-1.1 (2.9)

**Differences between Discharge and 3-mo. follow-up (Δ = 3 mo. post-OP - Discharge)**				
	TKA patients (n = 71), mean (SD)		THA patients (n = 105), mean (SD)	
Frailty	< 3	≥ 3	< 3	≥ 3
	*n* = 49	*n* = 27	*n* = 61	*n* = 48
Barthel Index	1.0 (5.8)	4.4 (10.6)	1.6 (3.5)	9.7 (19.0)*
MMSE	0 (1.6)	0.3 (1.5)	0 (1.5)	0.6 (3.7)
GDS	0.2 (1.3)	0.1 (1.7)	-0.2 (1.2)	0.1 (2.4)
NRS	0.1 (0.4)	0 (1.3)	-0.1 (0.5)	-0.2 (0.7)
Frailty	-0.1 (0.8)	-0.3 (1.1)	-0.3 (0.8)	-0.8 (1.1)*
SPPB	1.3 (1.5)	1.1 (1.7)	0.8 (1.8)	1.6 (2.0)
Grip strength	-0.4 (2.0)	-0.4 (4.5)	-0.3 (3.2)	1.0 (4.4)
Gait Speed	0.2 (0.2)	0.1 (0.2)	0.1 (0.2)	0.2 (0.2)*
Medication, n	-1.2 (1.4)	-0.8 (1.1)	-0.6 (1.4)	-1 (1.2)*

*TKA* Total knee arthroplasty, *THA* Total hip arthroplasty, *SD* Standard deviation, *MMSE* Mini Mental Status Examination, *GDS* Geriatric Depression Scale,* NRS*  Nutritional Risk Screening, *Frailty* Fried’s frailty criteria,* SPPB* Short Physical Performance Battery, Grip strength in kg, 5-meter gait speed test in m/s

* = p < 0.05

Mann-Whitney-U test

Patients in the knee group with higher physical performance (SPPB ≥ 7) benefited significantly less in terms of NRS during the rehabilitation phase than patients in lower SPPB categories (SPPB < 7). The same was found for participants in the hip group in terms of improvements in the SPPB. Also grouped on the basis of physical performance, significantly worse improvements in the Barthel Index and frailty were observed for THA patients with an SPPB of 7 or more points after discharge from rehabilitation up to 3 months after THA. This can be seen in detail in Table 6.

**Table 6:** Differences between start and end of rehabilitation as well as end of rehabilitation and 3 months after surgery based on SPPB

Differences between Admission and Discharge (Δ = Discharge - Admission)				
	TKA patients (n = 71), mean (SD)		THA patients (n = 104), mean (SD)	
SPPB	< 7	≥ 7	< 7	≥ 7
	*n* = 36	*n* = 39	*n* = 66	*n* = 43
Barthel Index	13.1 (9.8)	9.2 (9.8)	6.8 (15.9)	8.4 (11.6)
MMSE	0 (1.7)	0.2 (2.1)	-0.1 (4.2)	-0.2 (1.5)
GDS	-0.4 (1.5)	-0.1 (2.1)	-0.1 (2.3)	0.2 (1.3)
NRS	-0.5 (1.0)	-0.1 (0.4)*	-0.2 (0.7)	0 (0.6)
Frailty	-1.1 (1.4)	-0.7 (1.1)	-0.8 (1.4)	-0.8 (1.1)
SPPB	1.6 (2.4)	0.8 (1.5)	2.1 (2.8)	0.3 (2.2)*
Grip strength	0.2 (3.8)	0.5 (3.5)	0.6 (3.3)	-0.2 (3.4)
Gait Speed	0.2 (0.2)	0.3 (0.3)	0.2 (0.2)	0.2 (0.2)
Medication, n	-1.1 (2.3)	-1.1 (1.7)	-0.7 (2.9)	-1.5 (1.7)

**Differences between Discharge and 3-mo. follow-up (Δ = 3 mo. post-OP - Discharge)**				
	TKA patients (n = 71), mean (SD)		THA patients (n = 105), mean (SD)	
SPPB	< 7	≥ 7	< 7	≥ 7
	*n* = 36	*n* = 39	*n* = 66	*n* = 43
Barthel Index	3.0 (11.0)	1.5 (2.9)	8.5 (17.1)	3.3 (10.5)*
MMSE	0.3 (1.5)	-0.1 (1.6)	0.4 (4.2)	0.2 (1.2)
GDS	0.2 (1.7)	0.1 (1.1)	0.2 (2.2)	-0.2 (1.5)
NRS	0 (1.1)	0.1 (0.5)	-0.1 (0.7)	-0.1 (0.6)
Frailty	-0.2 (1.0)	0 (0.8)	-0.8 (1.1)	-0.3 (0.9)*
SPPB	1.5 (1.9)	0.9 (1.2)	1.5 (2.4)	1.0 (1.6)
Grip strength	-0.7 (3.6)	-0.1 (2.6)	0.7 (4.7)	0 (3.2)
Gait Speed	0.1 (0.2)	0.1 (0.3)	0.1 (0.2)	0.1 (0.2)
Medication, n	-1.1 (1.6)	-1.1 (1.1)	-0.6 (1.5)	-0.8 (1.2)

*TKA* Total knee arthroplasty, *THA *Total hip arthroplasty,* SD* Standard deviation, *MMSE* Mini Mental Status Examination, *GDS* Geriatric Depression Scale, *NRS* Nutritional Risk Screening,* Frailty*  Fried’s frailty criteria, *SPPB *Short Physical Performance Battery, Grip strength in kg, 5-meter gait speed test in m/s

* = p < 0.05

Mann-Whitney-U test

## Discussion

The aim of this study was to perform a comprehensive geriatric assessment of orthogeriatric patients after elective hip and knee replacement prior to rehabilitation, at the end of rehabilitation and 3 months after TJA, and to compare the results in order to draw conclusions about the development of CGA in this cohort of patients, which will grow rapidly in the coming years, immediately after rehabilitation but also in the subsequent course.

After completion of multimodal inpatient rehabilitation, the CGA showed significant improvements in activities of daily living, malnutrition, frailty, physical performance, gait speed and medication reduction in both the TKA and THA groups. With the exception of NRS and frailty in the TKA patients, the CGA also improved significantly 3 months postoperatively compared to discharge from rehabilitation. Differentiated according to the presence of frailty (Fried’s frailty criteria ≥ 3), both TJA groups showed significantly better improvements in frail participants than in robust or pre-frail patients. This suggests that obviously frail patients after TJA benefit particularly well from the effects of inpatient rehabilitation. This positive trend also continued in the further course after discharge from rehabilitation, at least for the frail participants in the THA group.

In the context of previous literature on intervention and reversal of frailty, this observation is easy to understand. For example, in the review by Kolle et al. most single and multi-component interventions to reverse frailty included physical activity [26]. Frail patients with OA after TKA and THA also demonstrated a significant improvement in frailty just a few days after surgery, while pre-frail patients initially deteriorated in this respect [9]. There appears to be particular potential for improving CGA after rehabilitation in patients with frailty.

Overall, there are very few studies on the course of CGA after elective TJA and inpatient rehabilitation.

Sonoda et al. used the CGA to investigate the effect of hospitalisation and long-term rehabilitation after TKA and THA. Although the assessment parameters used were essentially different, there were also numerous improvements in CGA at discharge from rehabilitation in both groups. Compared to our study, however, the number of patients included was significantly lower. Some participants were under 65 years of age and the majority were without multimorbidity. Frailty was not assessed and patients with cognitive impairment were excluded. Therefore, based on the patient characteristics, it must be assumed that although a CGA was used, it was not analysed exclusively in an orthogeriatric patient cohort. The observation period related only to admission and discharge from rehabilitation [27]. It is important to emphasise at this point that the convalescence of orthogeriatric patients after TJA does not end with discharge from inpatient rehabilitation, but that further functional improvements also occur in the subsequent course, as the CGA we carried out 3 months after surgery showed. A further reduction in medication is also possible in many cases, particularly by reducing or stopping analgesics.

This has also been demonstrated in previous studies. In their prospective study, de Groot et al. already described the positive effects of THA/TKA on physical function and activity after 3 and 6 months, albeit in younger and fitter patients [28]. Vissers et al. showed that patients who had undergone total hip and knee arthroplasty continued to improve their physical function and capacity even up to 4 years after the operation. Activities of daily living also improved up to 4 years after TJA. However, the number of participants included was significantly lower. In addition, this study did not include typical orthogeriatric patients, as patients over 80 years of age, non-independent persons, and patients with comorbidities other than OA were excluded [29].

Scott et al. analysed postoperative cognitive dysfunction (POCD) after total joint arthroplasty in a meta-analysis. The follow-up intervals considered were before discharge and 3–6 months after TJA. In the period immediately after surgery, there was data for a slight deterioration in general cognitive ability, but no evidence of deterioration 3–6 months after TJA. Considering the course of the MMSE, this is consistent with the CGA of Sonoda et al. and our results [30].

In 2020, Pfeufer et al. were able to show in their study based on a total number of 161 hip fracture patients that geriatric patients had a significantly better functional status after inpatient rehabilitation than patients without rehabilitation, both in the short and long term. The study also used the Barthel Index to assess participants’ functional status as part of the CGA. Without comparison to a control group, our results also show a significant improvement in the functional status of geriatric TJA patients, as measured by the Barthel Index, at discharge from inpatient rehabilitation and 3 months after surgery [14].

The main strength of this study is its prospective design with a large number of participants. Drop-out and loss to follow-up rates are very low for orthogeriatric patients, despite their vulnerability and often limited independence in daily activities. Not only the CGA between admission and discharge was compared, but also the CGA 3 months after TJA with discharge from rehabilitation. Furthermore, separate analyses were conducted for TKA and THA. Strict inclusion and exclusion criteria were used to ensure that only orthogeriatric patients were enrolled. The chosen CGA is well established both clinically and scientifically in geriatrics worldwide. The parameters used have been scientifically well researched and are characterised by high validity and reliability. Only the close interdisciplinary collaboration between orthopaedic surgeons and geriatricians in the SOG study enabled the specific application of CGA after TJA in the context of inpatient rehabilitation. Data analysis was performed externally and independently by the Department of Health Economics at the Technical University of Munich.

This study has some limitations. The study is part of the SOG trial and the analyses presented here were not originally planned. There is no control group for the evaluation. Strictly speaking, this would be necessary to determine the sole contribution of inpatient rehabilitation to the improvement in CGA. However, it seems unlikely that enough elderly, immobile and multimorbid patients who are willing to forgo routine inpatient rehabilitation paid for by their health insurance company could be recruited for a randomised controlled trial after elective TKA or THA. Participants may have completed orthopaedic or geriatric rehabilitation. Finally, in-house or external rehabilitation may result in different rehabilitation programmes for patients.

## Conclusion

The results of this study reveal that key components of the comprehensive geriatric assessment in orthogeriatric patients undergoing elective total hip and knee arthroplasty improve significantly after three weeks of inpatient rehabilitation and continue to improve up to three months after surgery. In addition, there is evidence that obviously frail patients benefit particularly well from the rehabilitation interventions. 

## Data Availability

The data are available on reasonable request from the corresponding author.

## Abbreviations

ADL: Activities of Daily Living
BMI: Body Mass Index
CCI: Charlson Comorbidity Index
CGA: Comprehensive geriatric assessment
ES: effect size
GDS: Geriatric Depression Scale
MMSE: Mini Mental Status Examination
mo.: month
n: number
NRS: Nutritional Risk Screening
OA: Osteoarthritis
POCD: Postoperative cognitive dysfunction
SD: Standard deviation
SPPB: Short Physical Performance Battery
THA: Total hip arthroplasty
TJA: Total joint arthroplasty
TKA: Total knee arthroplasty
WHO: World Health Organisation
